# Case Report: A multidisciplinary, protocol-driven pathway from recurrent pregnancy loss to live birth in an anti-Ro/SSA-positive primary Sjögren’s syndrome pregnancy with literature review

**DOI:** 10.3389/fimmu.2025.1702173

**Published:** 2026-01-16

**Authors:** Jing Zhang, Jianhong Chen, Sheng-Guang Li, Xiangyuan Liu

**Affiliations:** 1 Department of Rheumatology and Immunology, Peking University International Hospital, Beijing, China; 2 Reproductive Health and Infertility Clinic, Xinhai Maternity and Gynecology Hospital, Hefei, Anhui, China

**Keywords:** anti-Ro/SSA antibodies, congenital heart block, hydroxychloroquine, immunoglobulin therapy, neonatal lupus, pregnancy management, primary Sjögren’s syndrome, recurrent pregnancy loss

## Abstract

**Background:**

Maternal anti-Ro/SSA (Sjögren's syndrome-related antigen A) [± anti–La/SSB (Sjögren's syndrome type B antigen)] antibodies can lead to neonatal lupus, which may present most severely as autoimmune congenital atrioventricular block (CAVB). Although CAVB is uncommon (~ 2% of anti-Ro/SSA-positive pregnancies), once a complete block develops, spontaneous reversal is rare, and many affected infants require permanent pacemaker implantation. Consequently, prevention and early detection are critical components of management, particularly in pregnancies following previous antibody-mediated losses.

**Case presentation:**

A 34-year-old woman with primary Sjögren’s syndrome, high-titer anti-Ro/SSA and anti-La/SSB, and a history of five prior pregnancy losses—two early (biochemical, 7 weeks) and three midtrimester (16–21 weeks) complicated with severe fetal complete heart block (one demise, two terminations for hydrops)—presented for her sixth pregnancy. A multidisciplinary protocol was implemented, including hydroxychloroquine 400 mg/day, methylprednisolone (initially 6 mg/day, briefly increased to 32 mg/day at 14–16 weeks, then tapered to 8 mg/day), low-dose aspirin 50 mg/day, and fondaparinux 2.5 mg/day from ovulation throughout pregnancy, along with scheduled intravenous immunoglobulin (IVIG; 20 g at 4, 6 + 6, and 8 + 2 weeks; followed by 20 g/day × 3 at 14, 18, and 22weeks). From 14weeks, weekly fetal echocardiography with Doppler atrioventricular (AV)-interval monitoring (16–26weeks) remained normal. At 38 + 2weeks, a cesarean section delivered a female infant weighing 2,710 g and measuring 49 cm, with Apgar scores of 9/10. Neonatal telemetry/ECG showed sinus rhythm at 144 bpm without AV block. Echocardiography revealed a patent ductus arteriosus and a small atrial septal defect, with moderate pulmonary hypertension (SPAP 51 mmHg). Brain MRI and EEG were normal, and there were no cutaneous, hepatic, hematologic, or other features of neonatal lupus. Postpartum, the mother continued methylprednisolone 6 mg/day, hydroxychloroquine 400 mg daily, and enoxaparin 4,000 IU once daily for 4 weeks maintain disease suppression and thromboprophylaxis.

**Conclusion:**

In an anti-Ro/SSA-positive pregnancy at extreme risk, a prevention-first, protocol-driven approach—centered on hydroxychloroquine, judicious immunomodulation, and structured AV-interval surveillance—successfully averted CAVB and resulted in a pacemaker-free live birth. Minor cardiac lesions warrant ongoing follow-up; however, the absence of conduction disease underscores the clinical utility of this strategy in carefully selected, extreme-risk pregnancies.

## Introduction

Neonatal lupus results from the transplacental passage of maternal Immunoglobulin G (IgG) anti-Ro/SSA (often with anti-La/SSB). Its most severe manifestation is autoimmune congenital atrioventricular block (CAVB), which can develop abruptly in midgestation and is frequently irreversible once complete. Morbidity and mortality are substantial, and many affected infants ultimately require permanent cardiac pacing (1, 2).

Mechanistically, anti-Ro/SSA autoantibodies—particularly anti-Ro52—bind antigens exposed on apoptotic fetal cardiomyocytes, forming immune complexes that engage Fcγ-receptor-bearing macrophages, induce profibrotic cytokines, and replace atrioventricular nodal tissue with scar tissue. Inhibitory effects on fetal cardiac calcium channels may further slow conduction (3, 4). In contrast, anti-Ro60 antibodies are less specifically associated with this fibrotic injury (5). Transplacental IgG transfer accelerates from the late first trimester, and the clinical risk for antibody-mediated conduction delay clusters between 16 and 26 weeks—precisely the period most guidelines recommend for intensified surveillance (6).

Although the absolute risk of CAVB among anti-Ro/SSA-positive pregnancies is low (~ 2%) (2), recurrence after a previously affected fetus rises to 17%–20%, underscoring the need for prevention-first strategies and vigilant monitoring (6, 7). Hydroxychloroquine (HCQ) has emerged as the cornerstone of secondary prevention: in the prospective PATCH study, HCQ 400 mg/day started early in pregnancy reduced recurrent advanced block by > 50% versus historical rates, a finding reflected in guideline recommendations to treat anti-Ro/SSA-positive pregnancies with HCQ (8).

When fetal first- or second-degree AV block is detected, fluorinated corticosteroids (e.g., dexamethasone 4 mg/day) may be considered. However, treatment is not recommended for isolated third-degree block, as reversal is unlikely and maternal–fetal toxicity can accumulate (9, 10). Evidence for prophylactic intravenous immunoglobulin (IVIG) is mixed; the multicenter Prevention of Recurrence of Immune-mediated Diseases in Embryos (PRIDE) trial demonstrated that low-dose IVIG did not reduce recurrence, and thus routine use is not advised outside selected high-risk contexts (11). Accordingly, guidelines endorse serial fetal echocardiography—including Doppler-derived atrioventricular (AV)-interval assessment—weekly from ~ 16 to 26 weeks in mothers with prior neonatal lupus, to detect early conduction delay and enable timely intervention (12).

Primary Sjögren’s syndrome is a prototypic maternal autoimmune condition associated with the presence of anti-Ro/SSA antibodies. This report describes a protocol-driven, multidisciplinary pathway that translated mechanistic understanding and guideline recommendations into day-to-day perinatal decision-making, culminating in a term live birth without the need for neonatal pacemaker placement.

## Case presentation

### Patient information and obstetric history

A 34-year-old nulliparous woman with a 3-year history of primary Sjögren’s syndrome (pSS) was referred for management of her sixth pregnancy. She reported chronic xerostomia and intermittent arthralgia, without a history of systemic lupus erythematosus, renal disease, or cardiac disease. Objective ocular dryness was confirmed by a positive Schirmer’s test (≤ 5 mm/5 min). Unstimulated whole salivary flow was not performed due to patient refusal; however, she met the 2016 ACR/EULAR classification criteria based on anti-Ro/SSA positivity combined with objective ocular dryness (13). Family history was negative for congenital heart disease, autoimmune disease, or sudden cardiac death. She denied tobacco, alcohol, or illicit drug use.

Her obstetric history included five prior pregnancy losses: two early losses (one biochemical pregnancy and one miscarriage at 7 weeks) and three midtrimester losses (16–21 weeks), each complicated by severe immune-mediated fetal complete atrioventricular block confirmed by fetal echocardiography. In the first midtrimester pregnancy, the fetus died and delivery was induced; in the subsequent two pregnancies, termination was performed because of advanced hydrops despite preserved fetal heart motion. No structural fetal cardiac anomalies were identified in any of the earlier pregnancies.

### Preconception and early-gestation evaluation

Preconception disease activity was low. Serology confirmed high-titer anti-Ro/SSA (Ro52 and Ro60) and anti-La/SSB antibodies; Antinuclear antibody (ANA) testing was strongly positive, while anti-dsDNA antibodies were negative, and complement levels were normal. Antiphospholipid antibodies were negative on two occasions. Baseline maternal electrocardiogram (ECG) and echocardiogram were normal. The patient had been taking HCQ 400 mg/day continuously (including throughout her prior pregnancies, with good adherence).

### Preventive pharmacologic protocol

**Figure 1:**
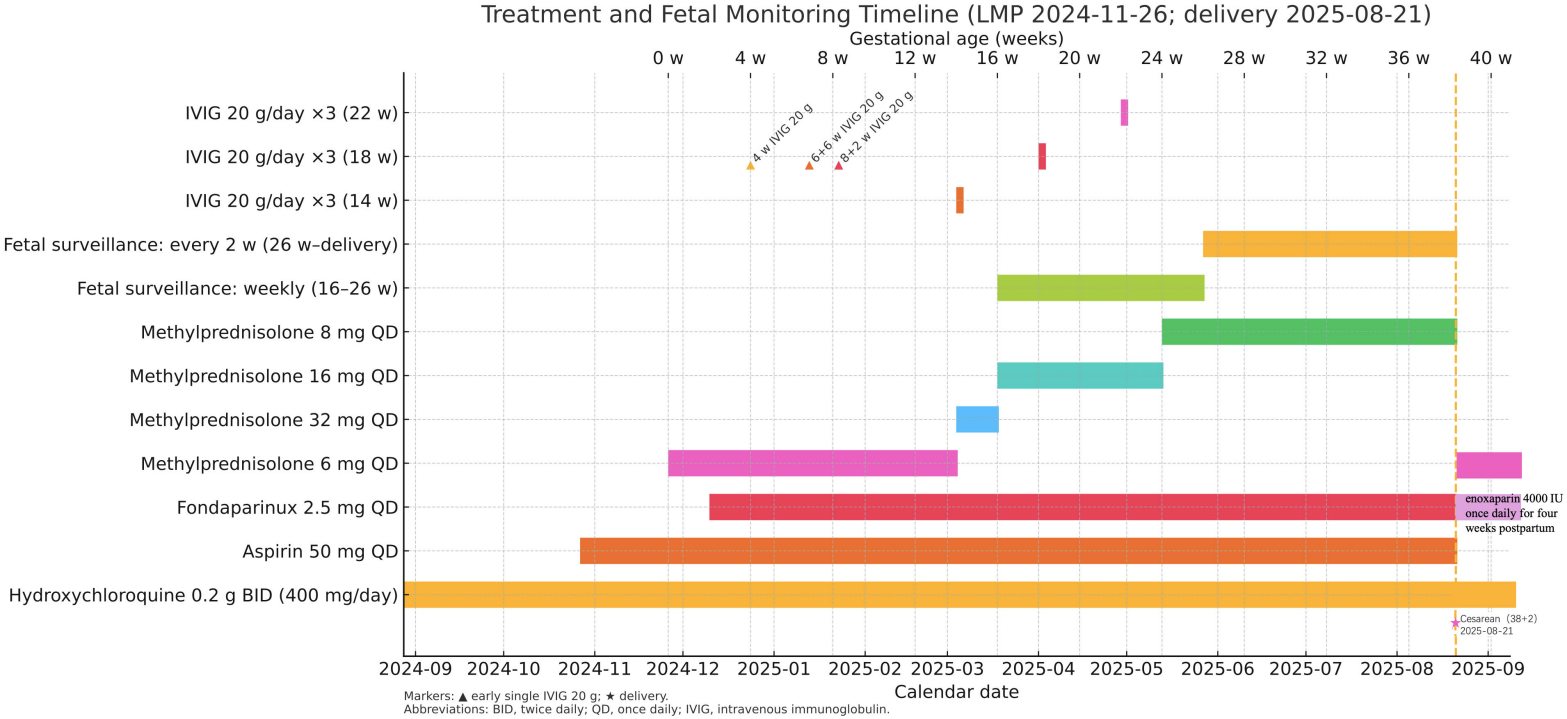
Preconception-to-postpartum timeline of preventive therapy in an anti-Ro/SSA-positive primary Sjögren’s pregnancy. Horizontal bars show the duration of therapies and surveillance from preconception through pregnancy, with annotations for continuation of key treatments postpartum. Hydroxychloroquine 0.2 g twice daily (400 mg/day) was continued from ≥ 3 months preconception through delivery; aspirin 50 mg once daily from ≥ 1month preconception through delivery; and fondaparinux 2.5 mg once daily from ovulation through delivery. Methylprednisolone followed a predefined schedule: 6 mg/day until 14 weeks; 32 mg/day at 14–16 weeks; 16 mg/day at 16–24 weeks; then 8 mg/day from 24 weeks to delivery. IVIG (10%, 20 g/infusion) was administered as single doses at 4, 6 + 6, and 8 + 2 weeks (▴), and as 3-day cycles (20 g/day × 3) at 14, 18, and 22 weeks (bars). Fetal surveillance comprised weekly echocardiography with Doppler-derived AV-interval monitoring from 16 to 26 weeks, then every 2 weeks until delivery. The vertical dashed line and star (★) mark cesarean delivery at 38 + 2 weeks (21 August 2025). The top axis displays gestational age (weeks), and the bottom axis shows calendar dates; LMP was 26 November 2024. Postpartum, methylprednisolone 6 mg/day was continued, and low molecular weight heparin (LMWH) prophylaxis was administered for 4 weeks.

In this index pregnancy, hydroxychloroquine 400 mg/day was continued throughout pregnancy. Glucocorticoids followed a predefined taper: methylprednisolone 6 mg/day before 14weeks, escalated to 32 mg/day at 14–16 weeks, reduced to 16 mg/day from 16 to 24weeks, then maintained at 8 mg/day from 24 weeks to delivery. Low-dose aspirin 50 mg once daily and fondaparinux 2.5 mg once daily were administered from ovulation throughout pregnancy. Fondaparinux was selected because the patient had previously developed significant and persistent injection-site reactions with enoxaparin during earlier pregnancies, making daily Low Molecular Weight Heparin (LMWH) difficult to tolerate. As her antiphospholipid antibodies were repeatedly negative and there was no indication for therapeutic-intensity anticoagulation, prophylactic-dose fondaparinux was considered an acceptable alternative after multidisciplinary discussion, and she tolerated it well without adverse events. IVIG was given as a 10% preparation, 20 g per infusion: single infusions at 4, 6 + 6, and 8 + 2weeks, and three-day cycles (20 g/day × 3) at 14, 18, and 22 weeks (**Figure 1**). Infusions were well tolerated aside from transient post-infusion headache and fatigue; there were no thrombotic or hemolytic events.

### Structured fetal cardiac surveillance

Fetal echocardiography began at 14 weeks, weekly from 16 to 26 weeks, then every 2 weeks until delivery, with a standardized worksheet: rhythm and 1:1 AV association, Doppler-derived mechanical AV interval (mitral inflow to aortic outflow), M-mode atrial/ventricular motion, systolic function, valvular competence, and hydrops survey. Throughout the high-risk window, studies consistently showed sinus rhythm with 1:1 conduction, non-prolonged AV interval, normal systolic function, no valvular regurgitation, and no hydrops. Second-trimester anatomy scans confirmed normal cardiac structure and normal fetal growth. Throughout the pregnancy, the mother remained clinically stable, with no autoimmune flares and all laboratory assessments within normal ranges.

### Third-trimester management and delivery

Given persistently normal fetal studies, the echocardiographic surveillance was decreased to every 2 weeks after 26 weeks, with routine obstetric monitoring maintained. On 21 August 2025 (38 + 2 weeks), the patient underwent cesarean delivery for obstetric indications. A female neonate weighed 2,710 g and measured 49 cm, with Apgar scores of 9 and 10 at 1 and 5 min, respectively. Intraoperative and immediate postoperative courses were uneventful.

### Immediate neonatal assessment and hospital course

Continuous telemetry/ECG demonstrated a heart rate of 144 bpm in sinus rhythm, with no evidence of atrioventricular block. Transthoracic echocardiography (performed on days 1–2) revealed a patent ductus arteriosus (PDA) and a small atrial septal defect (ASD), with an estimated systolic pulmonary artery pressure of 51 mmHg, consistent with moderate pulmonary hypertension. Biventricular size and systolic function were otherwise normal, and no pericardial effusion was observed. Cranial MRI and EEG were unremarkable. There were no cutaneous, hepatic, hematologic, or other manifestations of neonatal lupus. The infant remained clinically stable and was discharged with pediatric cardiology follow-up for monitoring of PDA/ASD and pulmonary pressures. At the 2-month follow-up, echocardiography demonstrated closure of the patent ductus arteriosus and resolution of pulmonary hypertension; the small atrial septal defect persisted but was hemodynamically insignificant. The infant has maintained a normal sinus rhythm, with ongoing cardiology surveillance planned to monitor the atrial defect and detect any potential late-onset conduction abnormalities.

### Postpartum maternal and neonatal course

Postoperatively, the mother continued methylprednisolone 6 mg daily, HCQ 400 mg/day, and enoxaparin 4,000 IU once daily for 4 weeks postpartum. Enoxaparin was selected because its shorter duration reduces the cumulative risk of injection-site intolerance, and LMWH remains the standard agent with the most extensive safety data in the puerperium, when thrombosis risk is highest. Laboratory tests showed ANA at 1:1,000 and strongly positive anti-Ro/anti-La antibodies, with persistently negative antiphospholipid antibodies and normal liver/renal function. Postoperatively, leukocytosis (WBC 17.36 × 10^9^/L) and elevated C-reactive protein (CRP) 28.59mg/L were attributed to the inflammatory response, with no clinical signs of infection. Both mother and neonate were discharged in good condition, with routine rheumatology and pediatric cardiology follow-up. At the 6-week postpartum evaluation, the mother remained in remission, with no pSS flare, while continuing HCQ therapy. A prednisone taper was initiated (from 6 to 4 mg daily, with planned discontinuation). She completed 4 weeks of postcesarean enoxaparin prophylaxis without complications. Follow-up laboratory tests demonstrated resolution of postoperative leukocytosis and CRP elevation, and persistently negative antiphospholipid antibodies.

At the infant’s 2-month pediatric cardiology follow-up, serial ECGs continued to demonstrate normal sinus rhythm with no conduction abnormalities. Follow-up echocardiography showed complete closure of the patent ductus arteriosus and resolution of the previously noted pulmonary hypertension, with only a small residual atrial septal defect that was hemodynamically insignificant. A long-term pediatric cardiology surveillance plan was established to monitor for any delayed-onset conduction abnormalities or structural sequelae.

## Discussion

### Principal finding

In a mother with pSS, high-titer anti-Ro/SSA and anti-La/SSB antibodies, and three prior midtrimester pregnancies complicated by severe immune-mediated atrioventricular block—including one fetal demise and two terminations for profound hydrops—a prevention-first, protocol-driven approach was implemented. This included continuous HCQ therapy, judicious corticosteroid use, adjunctive low-dose aspirin and prophylactic anticoagulation, scheduled IVIG at predefined gestational milestones, and structured weekly AV-interval monitoring from 16 to 26weeks. This strategy was associated with an uncomplicated term delivery of a female infant with normal cardiac conduction and no pacemaker requirement. Minor structural findings (PDA, small ASD) and moderate pulmonary hypertension were hemodynamically tolerated and remain under ongoing surveillance.

### Evidence anchoring the protocol

#### Hydroxychloroquine

Prospective data in anti-Ro/SSA-positive mothers with a previously affected child show that HCQ 400 mg/day, started early in pregnancy, reduces recurrent advanced block by > 50% versus historical rates, supporting its universal use for secondary prevention (8, 14, 15). Major guidelines conditionally recommend HCQ for anti-Ro/SSA (± La/SSB) pregnancies (16). Notably, anti-La/SSB antibodies rarely occur in isolation and are not sufficient by themselves to cause complete heart block (CHB) without anti-Ro/SSA (17); rather, their presence alongside anti-Ro/SSA is associated with only a modest increase in risk, reinforcing that anti-Ro52 is the principal pathogenic antibody in neonatal lupus (2). Continuous HCQ therapy before conception and throughout pregnancy, therefore, formed the foundation of the preventive strategy in this extremely high-risk patient.

#### Corticosteroids

Fluorinated steroids (e.g., dexamethasone) are indicated when first-/second-degree block is detected (18); therapy is not recommended for isolated third-degree block due to limited reversibility and potential maternal–fetal toxicity (19). In our patient, no conduction delay occurred that would necessitate high-dose dexamethasone. Nonetheless, a prophylactic methylprednisolone taper was administered, peaking at 32 mg/day in midgestation and subsequently tapered to 8 mg/day by delivery. This strategy aimed to control maternal disease activity and mitigate potential inflammation during the critical period. We acknowledge that 32 mg/day represents a significant dose; the term “low dose” refers to the maintenance dose of 6–8 mg/day outside the brief 2-week escalation. This approach was selected due to the patient’s extreme-risk profile.

#### IVIG

Replacement-dose IVIG did not significantly reduce CHB recurrence in the multicenter PRIDE trial (11), and the PITCH trial (20) similarly reported no clear benefit; therefore, routine prophylactic IVIG is not recommended. In this ultra-high-risk patient (three prior CHB-affected pregnancies), we elected to administer IVIG as an individualized, compassionate strategy despite the mixed evidence, after discussing the uncertainty of benefit with the patient.

#### Structured surveillance

Weekly fetal echocardiography with Doppler-derived AV-interval assessment from 16 to 26weeks is recommended for mothers with a history of neonatal lupus/CHB to detect early conduction delays and enable timely intervention (11, 21, 22). In this case, surveillance remained normal throughout the monitoring period.

### Why this outcome matters

After an affected gestation, the recurrent risk of immune-mediated CHB is substantially higher than the 2% baseline in anti-Ro/SSA pregnancies, and most survivors of complete block ultimately require pacemaker implantation. Achieving a pacemaker-free outcome by preventing *in utero* progression is therefore clinically meaningful and aligns with a prevention-first paradigm.

### Interpreting the neonatal findings

The neonate exhibited a PDA and a small ASD, accompanied by moderate pulmonary hypertension (SPAP ~ 51 mmHg), while conduction and normal ventricular function remained normal. Population studies indicate modestly increased odds of septal defects/PDA in offspring of mothers with systemic autoimmune disease; in individual cases, transitional circulatory physiology and intracardiac shunts may contribute to transient pulmonary pressure elevation (23, 24). Importantly, pulmonary hypertension is typically reported in the context of severe cardiac neonatal lupus (e.g., CHB/cardiomyopathy) (25–27). In this case, the absence of conduction abnormalities and preserved ventricular function support a non-Neonatal Lupus Erythematosus (NLE) mechanism, with anticipated improvement on follow-up.

We have arranged serial pediatric cardiology follow-ups to monitor for any delayed-onset conduction issues, acknowledging that maternal anti-Ro/La antibodies can persist in the infant’s circulation for several months postpartum. To date, the infant has shown no evidence of conduction delay, and the observed improvements in PDA closure, pulmonary pressures, and cardiac function support a benign, non-NLE-related course.

### Maternal postpartum considerations

Continuation of HCQ throughout pregnancy and into the postpartum period is standard practice to reduce disease flares. Given the patient’s established pSS and the recent immunological and physiological stress of pregnancy, a low dose of prednisone was maintained immediately postpartum to minimize flare risk, with a predefined tapering plan. Although HCQ alone is typically sufficient for long-term maintenance in pSS, a cautious transitional period before steroid withdrawal was preferred, with the option to switch to a steroid-sparing immunosuppressant if needed. LMWH thromboprophylaxis was continued for 4 weeks postpartum due to cesarean delivery and the transient postpartum hypercoagulable state (28). Postoperative leukocytosis/CRP elevation resolved without evidence of infection. The antenatal use of fondaparinux and the postpartum switch to enoxaparin were individualized decisions, based on prior intolerance to LMWH during pregnancy, negative antiphospholipid antibody status, and the stronger evidence supporting LMWH as the preferred agent for postpartum thromboprophylaxis.

### Strengths and limitations

Strengths of this report include the exceptionally high-risk baseline—three prior midtrimester pregnancies affected by CHB, including one fetal demise and two terminations for hydrops—the use of a predefined, protocol-driven pathway with clear escalation rules, and systematic documentation of fetal conduction status through weekly AV-interval monitoring during the critical 16–26-week period. The normal neonatal conduction outcome, combined with short-term postnatal follow-up, provides valuable clinical insight into the management of extreme-risk anti-Ro/SSA pregnancies.

Limitations are inherent to single-case reports. It remains unclear which specific intervention, if any, was pivotal in averting CHB in this pregnancy. In particular, the contributions of IVIG and the brief midgestation corticosteroid escalation cannot be isolated, and their prophylactic use remains unproven in clinical trials. Consequently, these components of the regimen should not be routinely adopted without compelling indications. This case should be interpreted as a deliberate deviation from standard practice under extraordinary clinical circumstances, rather than as evidence supporting a generalized strategy. Further studies are needed to clarify the relative importance of individual preventive interventions.

### Practice points

(1) Start HCQ preconception/early pregnancy in all anti-Ro/SSA (± La) pregnancies, particularly following a previously affected gestation; (2) plan weekly fetal echocardiography with AV-interval tracking from 16 to 26weeks of gestation; (3) escalate promptly to dexamethasone upon detection of first- or second-degree block; avoid steroids in cases of established complete block; (4) ensure coordinate multidisciplinary care and preplanned peripartum neonatal cardiology support; (5) counsel families regarding pacing expectations, while emphasizing that preventive strategies can alter disease trajectory.

### Patient perspective

“After losing five pregnancies, I was scared to try again. The specialized care plan gave me a bit of hope each week. The constant monitoring was stressful but also reassuring—I knew any problem would be caught immediately. Working with a team of doctors from different specialties made me feel supported. Hearing my baby’s heartbeat week after week, and then finally holding her, was an indescribable joy. I am so grateful for the team’s dedication. This successful birth has been life-changing, and I hope my story can help other women in similar situations find hope”.

## Conclusion

A coordinated, prevention-first pathway—HCQ-centered immunomodulation, predefined escalation, and structured AV-interval surveillance—transformed a history of recurrent, antibody-mediated fetal losses into a pacemaker-free term live birth. Minor cardiac lesions warrant follow-up, but the absence of conduction disease highlights the clinical utility of this approach in similarly extreme-risk cases.

## Data Availability

The original contributions presented in the study are included in the article/supplementary material. Further inquiries can be directed to the corresponding authors.
